# Investigating the impact of surgical masks on behavioral reactions to facial emotions in the COVID-19 era

**DOI:** 10.3389/fpsyg.2024.1359075

**Published:** 2024-04-04

**Authors:** Martina Montalti, Giovanni Mirabella

**Affiliations:** ^1^Department of Clinical and Experimental Sciences, University of Brescia, Brescia, Italy; ^2^IRCCS Neuromed, Pozzilli, Italy

**Keywords:** behavioral control, task-relevance, facial expressions, emotion, surgical mask, COVID-19, Go/No-go

## Abstract

**Introduction:**

The widespread use of surgical masks during the COVID-19 pandemic has posed challenges in interpreting facial emotions. As the mouth is known to play a crucial role in decoding emotional expressions, its covering is likely to affect this process. Recent evidence suggests that facial expressions impact behavioral responses only when their emotional content is relevant to subjects’ goals. Thus, this study investigates whether and how masked emotional faces alter such a phenomenon.

**Methods:**

Forty participants completed two reaching versions of the Go/No-go task in a counterbalanced fashion. In the Emotional Discrimination Task (EDT), participants were required to respond to angry, fearful, or happy expressions by performing a reaching movement and withholding it when a neutral face was presented. In the Gender Discrimination Task (GDT), the same images were shown, but participants had to respond according to the poser’s gender. The face stimuli were presented in two conditions: covered by a surgical mask (masked) or without any covering (unmasked).

**Results:**

Consistent with previous studies, valence influenced behavioral control in the EDT but not in the GDT. Nevertheless, responses to facial emotions in the EDT exhibited significant differences between unmasked and masked conditions. In the former, angry expressions led to a slowdown in participants’ responses. Conversely, in the masked condition, behavioral reactions were impacted by fearful and, to a greater extent, by happy expressions. Responses to fearful faces were slower, and those to happy faces exhibited increased variability in the masked condition compared to the unmasked condition. Furthermore, response accuracy to masked happy faces dramatically declined compared to the unmasked condition and other masked emotions.

**Discussion:**

In sum, our findings indicate that surgical masks disrupt reactions to emotional expressions, leading people to react less accurately and with heightened variability to happy expressions, provided that the emotional dimension is relevant to people’s goals.

## 1 Introduction

Facial expressions represent a form of non-verbal communication that can complement and enhance verbal communication, providing important cues about the emotional states, needs, and intentions of others, enabling us to manage and coordinate our social interactions. Thus, the ability to perceive and interpret facial expressions is a crucial aspect of social cognition, allowing individuals to respond appropriately in social contexts.

While there is evidence indicating that emotional stimuli affect motor behavior, consensus on the underlying mechanisms remains elusive (Mancini et al., 2020; Montalti and Mirabella, 2023). The prevailing theoretical framework for interpreting the effect of emotional stimuli on behavioral reactions is the motivational model (Bradley et al., 2001; Lang and Bradley, 2010). This model is built on the notion that, unlike most stimuli, emotional ones cannot be filtered by selective attention, and thus, they trigger automatic responses promoting our survival (Lang et al., 2000; Vuilleumier, 2005). In contrast, the appraisal theories of emotion (Moors and Fischer, 2019; Scherer and Moors, 2019) argue against the existence of attentional bias toward emotionally laden stimuli and assert that behavioral reactions to such stimuli vary depending on the situational context and the individual’s specific goals. In this context, a specific stimulus does not possess an inherently negative or positive value in absolute terms. The following example clarifies the concept. Suppose a normal person encounters a spider. She/he will likely have a fearful reaction, aiming to avoid it. Conversely, an entomologist, actively seeking spiders for his/her studies, will react joyfully, entailing an approach-oriented behavior. The appraisal theories received strong support from a recent series of studies that employed an innovative Go/No-go paradigm developed by Mirabella and colleagues (Mirabella, 2018; Mancini et al., 2020, 2022; Calbi et al., 2022; Mirabella et al., 2023; Montalti and Mirabella, 2023). In this experimental design, the same group of participants is engaged in two versions of the Go/No-go task, differing only in the instruction given to participants. In the emotional version (the Emotional Discrimination Task, EDT), participants have to move when they see an emotional face and withhold the response when it is neutral, or vice versa. In the control version, participants were instructed to respond according to other images’ features, such as the poser’s gender (Mirabella, 2018; Mancini et al., 2020, 2022; Mirabella et al., 2023; Montalti and Mirabella, 2023) or the t-shirt’s color (Calbi et al., 2022). Overall, Mirabella and colleagues’ findings unveiled that emotional valence exerted a significant influence on motor planning (Mirabella, 2018; Mancini et al., 2020; Mirabella et al., 2023; Montalti and Mirabella, 2023), and inhibitory control (Calbi et al., 2022; Mancini et al., 2022), provided that this stimulus dimension is relevant for task instructions, i.e., in the EDT. Their findings suggest that threatening stimuli, such as angry and fearful expressions, hold subjects’ attention stronger than happy faces, but only when the emotional connotation of the stimuli aligns with the participants’ goals. This phenomenon was named task-relevant effect.

Over the past three years, the outbreak of the COVID-19 pandemic has necessitated the widespread use of surgical masks. This has posed a novel challenge for recognizing others’ expressions as the masks cover the mouth region, one of the most relevant features for identifying emotional expressions (Engbert and Kliegl, 2003; Smith et al., 2005; Eisenbarth and Alpers, 2011; Bodenschatz et al., 2019). It is generally acknowledged that the mouth mainly contributes to the recognition of happiness, disgust, and surprise, while the eyes are deemed more relevant for detecting sadness and anger (Schurgin et al., 2014). The literature is discordant on fearful expressions recognition. Some studies argue for a substantial role of the eyes (Whalen et al., 2001; Morris et al., 2002; Adolphs et al., 2005), while others sustain that mouth and eyes provide equal contributions to the identification of fear (Eisenbarth and Alpers, 2011). Several previous studies have examined the impact of surgical masks on the identification of emotional faces and found that, as expected, healthy people do not show any impairments in recognizing anger, but they were impaired in recognizing happiness (Calbi et al., 2021; Marini et al., 2021; Grahlow et al., 2022; Proverbio and Cerri, 2022; Rinck et al., 2022; Ventura et al., 2023). Nevertheless, this evidence does not offer insights into how we respond to emotional faces, as the visual signals used for guiding motor actions are processed differently from those involved in perception (Goodale, 2014). To fill this gap, in the current study, we assessed the impact of masks on behavioral reactions elicited by three emotional facial expressions (angry, happy, and fearful faces). We chose such emotional expressions because we studied them previously using the above-described paradigm. Our primary objective was to assess whether the ask-relevance effect observed in unmasked faces persisted in the presence of surgical masks. In other words, we wondered whether healthy persons, in addition to their ability to recognize emotional expressions explicitly, were still able to implicitly use visual information to respond appropriately to such stimuli.

## 2 Materials and methods

### 2.1 Participants

Notably, 40 healthy volunteers participated in the present study (20 females; mean ± standard deviation age = 24.5 ± 3.6, range = 19.7–32.9). The sample size was determined in advance using G*Power 3.1.9.4 (Faul et al., 2009) using repeated-measure ANOVA. The input variables for such calculation were taken from previously published data (Mirabella, 2018; Mancini et al., 2020; Montalti and Mirabella, 2023; effect size = 0.15, alpha = 0.05, power = 0.80, number of measures = 12, correlations among repeated measures *r* = 0.5, and non-sphericity correction *e* = 0.8). The estimated minimum sample size was 37. All participants had normal or corrected-to-normal visual acuity and were naïve about the study’s purpose. None of the participants had a history of psychiatric or neurological disorders.

Before the experimental session, we assessed the presence of alexithymia, which is a condition characterized by difficulty or inability to identify, express, and communicate one’s own emotions. It also makes it difficult for people to understand and respond to other people’s emotions, leading to difficulties in social relationships. Consequently, we considered the alexithymia as an exclusion criterion. As a screening tool, we administered the Italian version of the Toronto Alexithymia Scale (TAS-20; Bagby et al., 1994; Bressi et al., 1996). This scale consisted of 20 statements, and participants were required to indicate their level of agreement using a 5-point Likert scale. Participants were included in the study only if their TAS-20 total score was smaller than the alexithymia threshold, i.e., 61.

The study received approval from the local ethical committee “ASST Spedali Civili” di Brescia (protocol number 4452) and was conducted in accordance with the principles outlined in the Declaration of Helsinki (2013). Before the study’s commencement, all participants provided written informed consent to participate.

### 2.2 Stimuli

**Figure 1:**
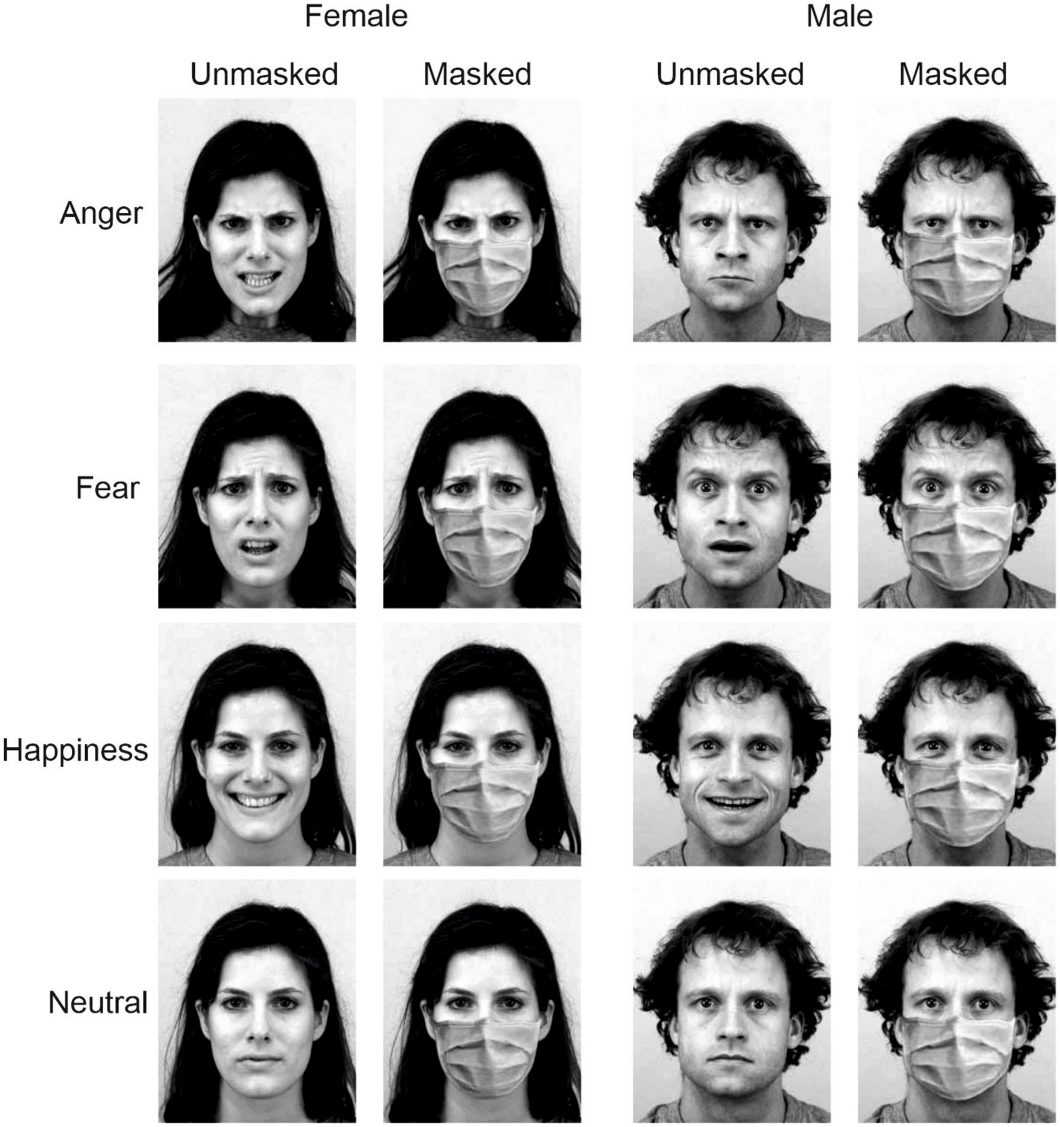
Examples of the experimental stimuli depict angry, fearful, happy, and neutral facial expressions of a man and a woman. Stimuli were taken from the Karolinska Directed Emotional Faces (KDEF; Lundqvist et al., 1998; https://kdef.se/home/aboutKDEF). Pictures were modified, adding a surgical mask to obtain a masked and an unmasked version of each stimulus’. Stimuli were reproduced and adapted with permission (see https://kdef.se/faq/using-and-publishing-kdef-and-akdef).

Stimuli were angry, fearful, happy, and neutral facial expressions interpreted by eight actors (four females) taken from the Karolinska Directed Emotional Faces (KDEF; Lundqvist et al., 1998). Using Photoshop software (version 2020, Adobe Systems Inc., San Jose, United States), we added a surgical mask to each stimulus to obtain a masked and an unmasked version of each stimulus. Thus, we created 64 different stimuli (Figure 1).

At the end of the experimental sessions, two questionnaires were administered to evaluate the arousal and valence of each stimulus. In the arousal questionnaire, participants had to evaluate each stimulus using a 7-point Likert scale ranging from “1 = not at all arousing” to “7 = very much arousing.” The valence questionnaire had a similar structure, but the Likert scale ranged from “1 = negative” to 7 = positive.” The middle point, i.e., 4, was labeled as neutral.

For both valence and arousal and each participant, we calculated the average ratings of each experimental condition. Such a procedure was conducted separately for each emotion and face with and without surgical masks. Although both arousal and valence ratings did not follow a normal distribution according to the Shapiro–Wilk test, we opted to use parametric ANOVA as our sample size was larger than 30 participants (Fagerland, 2012). This large sample size satisfies the requirements of the central limit theorem, making the parametric approach robust even in cases of deviations from the normal distribution (Rasch and Guiard, 2004). Both ANOVAs had two within-participant factors: Emotion (four levels: anger, fear, happiness, and neutral) and Mask (two levels: masked and unmasked).

The analysis of valence ratings revealed a significant main effect of Emotion (*F*(1.81, 70.43) = 597.68, *p* < 0.001, η^2^ₚ = 0.94). Post-hoc comparison with Bonferroni correction indicated that all emotions differed from one another (all *p_s_* < 0.0001). In particular, happy facial expressions (5.81 ± 1.06) were rated significantly more positive-valenced than angry (1.80 ± 0.86), fearful (2.35 ± 0.91), and neutral expressions (3.81 ± 0.72). Furthermore, neutral faces were judged more positive than angry and fearful expressions, while fearful expressions were considered more positive than angry ones. There was also a significant main effect of Mask (*F*(1, 39) = 40.66, *p* < 0.001, η^2^ₚ = 0.51), indicating that unmasked faces (3.52 ± 1.97) were rated more positive in valence than masked faces (3.36 ± 1.59). Finally, the ANOVA showed a significant interaction effect between Emotion and Mask (*F*(2.41, 93.85) = 65.08, *p* < 0.001, η^2^ₚ = 0.62). Post-hoc comparison with Bonferroni correction revealed that unmasked happy faces were rated more positively (6.29 ± 0.86) than masked ones (5.32 ± 1.03; *p* < 0.0001). Also, it revealed that within both masked and unmasked conditions, all emotions differed from each other (all *p_s_* < 0.0001). In both cases, these differences followed the same pattern observed in the main effect of Emotion.

The analysis of arousal ratings showed a significant main effect of Emotion (*F*(1.87, 72.82) = 79.67, *p* < 0.001, η^2^ₚ = 0.67). Post-hoc comparisons with Bonferroni correction revealed that neutral faces (2.12 ± 1.21) were rated less arousing compared to happy (3.69 ± 1.67), fearful (4.49 ± 1.47), and angry expressions (4.39 ± 1.48; all *p_s_* < 0.0001). Furthermore, happy expressions were rated significantly less arousing than both angry and fearful faces (*p_s_* < 0.0001), while angry and fearful faces did not differ significantly from each other (*p =* 1.00). We also found a main effect of Mask (F(1, 39) = 76.17, *p* < 0.001, η^2^ₚ = 0.66) because masked faces (3.32 ± 1.64) were perceived as less arousing than unmasked ones (4.03 ± 1.77). Finally, the analysis unveiled a significant interaction between Emotion and Mask (*F*(2.76, 107.64) = 21.01, *p* < 0.001, η^2^ₚ = 0.35). Post-hoc comparison with Bonferroni correction showed that the mask affected all emotions. But the neutral ones. In fact, masked expressions were rated less arousing than their unmasked counterparts (*p_s_* < 0.0001). Furthermore, within the masked expressions, all emotions differed (*p_s_* < 0.0001) except anger and fear. Differently, within the unmasked faces, only neutral expressions differed from all other emotions (all *p_s_* < 0.0001).

### 2.3 Experimental apparatus and procedure

**Figure 2:**
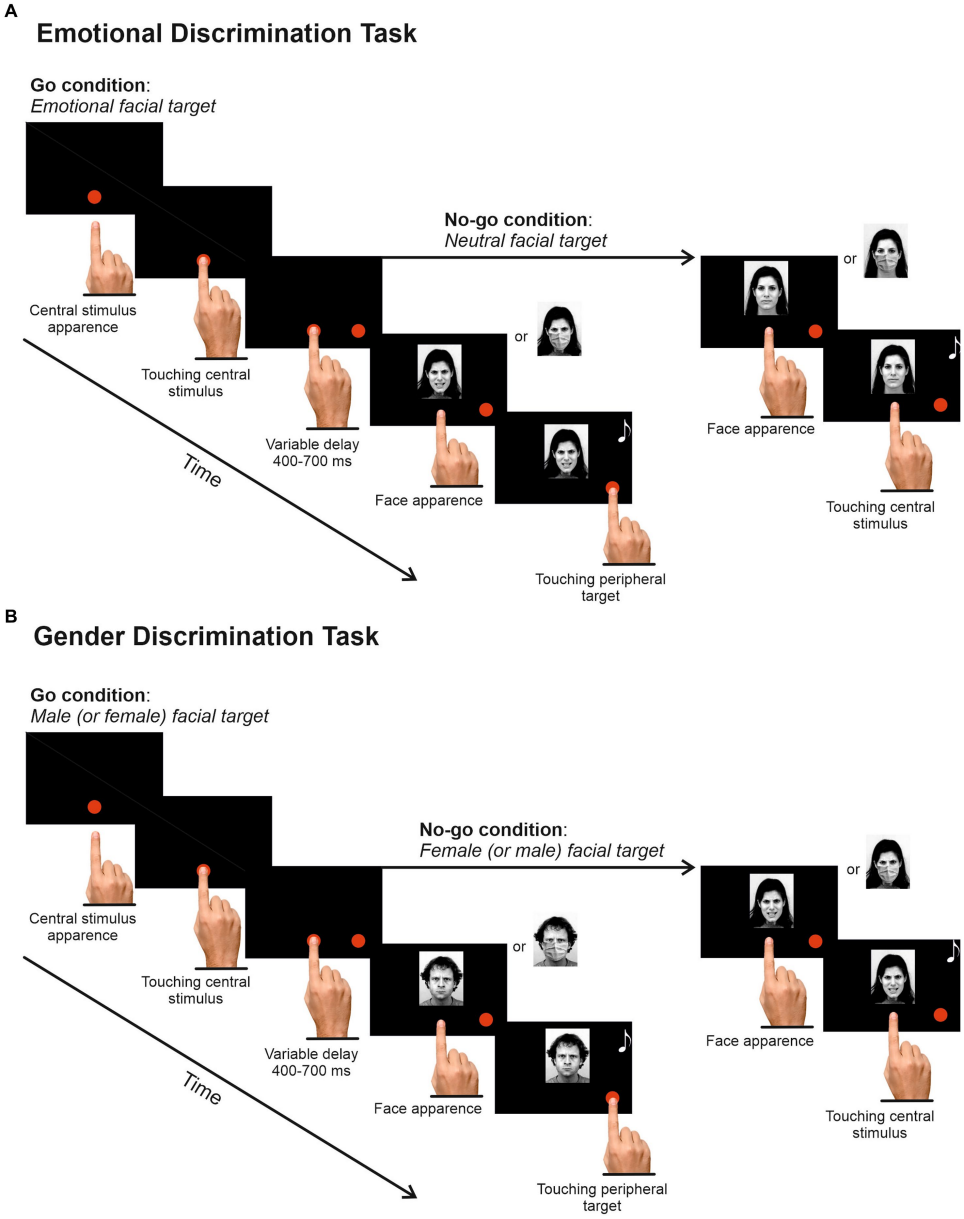
Experimental Design. **(A)** Emotional Discrimination Task. Each trial started with the appearance of a red dot at the center of the screen. Participants were instructed to touch and hold it for a random delay of 400–700 ms. Then, a peripheral red dot appeared on the right side of the screen, followed by an image depicting a face with angry, fearful, happy, or neutral expressions. Participants had to reach and hold the peripheral target when the face expressed an emotion (67%) and refrain from moving when the face had a neutral expression (33%). **(B)** The Gender Discrimination Task had the same structure, but participants had to respond or refrain from responding according to the poser’s gender. Half of the participants were instructed to respond to the male target and to withhold their response to the female target, and vice versa for the other half. The task order was counterbalanced across participants, and the experimental conditions were randomized in each task. Stimuli were reproduced and adapted with permission (see https://kdef.se/faq/using-and-publishing-kdef-and-akdef).

Participants were tested in a dimly lit and soundproofed room, using a 17-inch PC monitor where visual stimuli were displayed. The PC monitor was linked to a touch screen (MicroTouch; sampling rate 200 Hz) for monitoring touch position. The timing of the stimulus presentation was synchronized with the monitor’s refresh rate. Behavioral responses and stimulus presentation were controlled by Cortex, a non-commercial software package developed at NIH.^1^ In one experimental session, participants performed two different versions of the Go/No-go task in a counterbalanced fashion. In the EDT (Figure 2A), participants were instructed to reach a central red dot (2.43 cd/m^2^, diameter 2.8 cm or 4 dva) appearing two centimeters below the center of the screen, with the index finger of their dominant hand, previously assessed using the Italian version of the Edinburgh Handedness test (Oldfield, 1971). When participants touched the central dot, a peripheral target appeared on the right side of the screen at an eccentricity of 8 cm or 11.3 dva. Participants were instructed to hold the central dot for a variable time (400–700 ms) until it disappeared and a picture of a face (go-stimulus) appeared above it. When the face had an emotional connotation (go trials; 66%), participants had to reach the peripheral target as quickly and accurately as possible, holding it for a variable time (300–400 ms). Conversely, when the face displayed a neutral expression (no-go trials; 34%), they were instructed to remain still and hold the index on the central position for a random time (400–800 ms).

For both go and no-go trials, the correct execution was signaled by acoustic feedback, followed by an intertrial interval lasting 800 ms. During this period, the screen was black. In the go trials, participants had a maximum time of 500 ms to respond. However, we allowed participants an extra 100 ms to avoid cutting the right tail of reaction times (RTs) distribution. The upper-RT limit was set to discourage participants from slowing their movement to easy stopping. When the participants’ response was between 500 and 600 ms, the go trial was signaled as an error and aborted (overtime reaching trials, see Mirabella et al., 2006). Nevertheless, overtime-reaching trials were included in the analyses, accounting for 5.95% of the total go trials. Overall, the EDT had 432 trials that were given in two blocks to allow rest if requested. Experimental conditions were randomized and balanced across blocks.

The Gender Discrimination Task (GDT; Figure 2B) was identical to the EDT except that participants were instructed to respond according to the stimuli’ gender, i.e., half of the participants had to respond to the presentation of female faces and to withhold their response to male faces, and vice versa for the other half. The GDT also had 432 trials delivered in two blocks (34% no-go trials). The experimental conditions were randomized and balanced. The overtime-reaching trials accounted for 3.17% of the total go trials.

### 2.4 Statistical analyses

The impact of emotions on behavioral responses was investigated by analyzing three indices, calculated separately for each participant and condition, i.e., RTs and movement times (MTs) of correct go trials and omission error rates (OERs). RTs are defined as the time between the go-signal and the movement onset. The mean RT was calculated excluding go trials with an RT that exceeded three standard deviations above and below the mean. This procedure resulted in excluding 0.53% of trials in the EDT and 0.53% in the GDT. MTs are measured as the time between the movement onset and the moment when participants touch the peripheral target. Finally, OERs are instances in which participants did not respond to the go-signal, keeping touching the central stimulus. OERs were computed as the ratio between the number of errors in a given condition and the overall number of trials in the same condition, multiplied by 100.

As the analyses of arousal ratings revealed significant differences within the masked condition, we included arousal as a between-participant factor to rule out the possibility that such dimension, rather than valence, could explain the effect. To this aim, we exploited the Delta Arousal index, which was computed using the Revised Standardized Difference Test (RSDT; Crawford and Garthwaite, 2005) that allowed us to evaluate whether the standardized difference between the ratings of one individual significantly deviated from the average difference of the all the other participants, considered as the control group. Since the RSDT assessed the differences only between two variables, but we have three emotions, we opted to collapse anger and fear ratings, as their arousal did not differ in the masked and unmasked conditions. We then converted the resulting *z*-values into percentiles to create two equally sized subgroups, i.e., participants with a Delta Arousal ranging from the 30th to the 70th percentile were categorized as the “low arousal” subgroup (10 females; age = 23.9 ± 3.4 years, range = 19.7–32.9), while those outside this range were assigned to the “high arousal” subgroup (10 females; age = 25.2 ± 3.7 years, range = 20.3–31.9).

Separately for RTs, MTs, and OERs, we performed a four-way mixed-design ANOVA [within-participants factors: Emotion (three levels: anger, fear, and happiness); Task (two levels: EDT and GDT); Mask (two levels: masked and unmasked); between-participants factor: Delta Arousal (two levels: high and low)]. Additionally, for each behavioral parameter, we performed a control analysis for the GDT go trials, as in these trials, neutral facial expressions were also shown. We used a three-way mixed-design ANOVAs [within-participants factors: Emotion (four levels: anger, fear, happiness, and neutral); Mask (two levels: masked and unmasked); between-participants factor: Delta Arousal (two levels: high and low)].^2^ The Shapiro–Wilk tests showed that RTs and MTs were normally distributed, while the OERs were not. However, we also employed parametric ANOVA for OERs because we had a large sample (Fagerland, 2012).

Post-hoc tests were corrected using Bonferroni, and we reported the effect sizes as partial eta-squared (*η^2^_p_*) or Cohen’s *d*. Finally, we computed the Bayes Factors to quantify the null hypothesis’ strength (BF10; Jarosz and Wiley, 2014), setting the prior odds to 0.707 (R package BayesFactor; Morey and Rouder, 2018). Values of BF_10_ > 3 and > 10 indicate moderate and strong support for the alternative hypothesis, respectively. Values of BF_10_ < 0.1 and < 0.33 indicate strong and substantial support for the null hypothesis, and values 0.33 < BF_10_ < 3 are inconsistent for any hypothesis. All statistical analyses were performed using R, version 4.2.3 (R Core Team, 2020).

## 3 Results

Table 1 reports the means and standard deviations for each experimental condition. The results of the three behavioral indices, i.e., RTs, MTs, and OERs, will be presented in single subsections.

**Table 1:** Means (M) and standard deviations (SD) of Go trial reaction times, movement times, and omission error percentages.

	Emotional Discrimination Task			Gender Discrimination Task		
	Masked	Unmasked	*M* ± *SD*	Masked	Unmasked	*M* ± *SD*
	*M* ± *SD*	*M* ± *SD*		*M* ± *SD*	*M* ± *SD*	
**Reaction times (ms)**						
Anger	385.65 ± 38.51	379.36 ± 37.69	**382.50 ± 37.99**	371.27 ± 35.77	369.48 ± 40.11	**370.38 ± 37.77**
Fear	384.11 ± 40.85	363.18 ± 37.42	**373.64 ± 40.33**	374.62 ± 37.41	368.37 ± 40.27	**371.50 ± 38.75**
Happiness	375.92 ± 48.50	363.97 ± 40.00	**369.95 ± 44.58**	370.55 ± 38.08	367.72 ± 40.52	**369.14 ± 39.10**
Neutral	–	–	–	372.78 ± 38.44	367.89 ± 37.85	**370.34 ± 37.99**
**M** ± **SD**	**381.89 ± 42.69**	**368.84 ± 38.79**		**372.15 ± 36.83**	**368.52 ± 39.97**	
**Movement times (ms)**						
Anger	338.16 ± 89.97	338.57 ± 88.57	**338.37 ± 88.70**	327.65 ± 76.68	325.90 ± 75.56	**326.78 ± 75.65**
Fear	339.07 ± 90.41	333.68 ± 83.92	**336.38 ± 86.72**	326.31 ± 74.84	327.91 ± 76.62	**327.11 ± 75.26**
Happiness	380.78 ± 89.70	336.96 ± 86.36	**358.87 ± 90.22**	324.90 ± 75.81	327.92 ± 76.82	**326.31 ± 75.84**
Neutral	–	–	–	325.96 ± 73.92	327.01 ± 73.02	**326.48 ± 73.01**
**M** ± **SD**	**352.67 ± 91.47**	**336.41 ± 85.60**		**326.29 ± 75.15**	**327.18 ± 75.70**	
**Omission errors percentages**						
Anger	7.34 ± 5.59	6.35 ± 6.24	**6.84 ± 5.91**	2.02 ± 2.82	2.61 ± 3.45	**2.32 ± 3.15**
Fear	7.08 ± 5.6	3.98 ± 5.12	**5.54 ± 5.55**	2.11 ± 2.3	2.92 ± 4.43	**2.52 ± 3.78**
Happiness	23.79 ± 11.8	4.51 ± 4.51	**14.15 ± 13.14**	2.55 ± 3.23	2.33 ± 3.18	**2.44 ± 3.19**
Neutral	–	–	–	2.60 ± 3.38	2.57 ± 3.15	**2.58 ± 3.25**
**M** ± **SD**	**12.73 ± 11.30**	**4.95 ± 5.39**		**2.23 ± 3.00**	**2.62 ± 3.70**	

For each behavioral parameter, the shaded regions indicate the averages for each emotional expression, collapsing across the Mask factor (vertical shaded regions), and the averages for the masked and unmasked conditions, collapsing across the emotional expressions (horizontal shaded regions).

### 3.1 Reaction times

**Figure 3:**
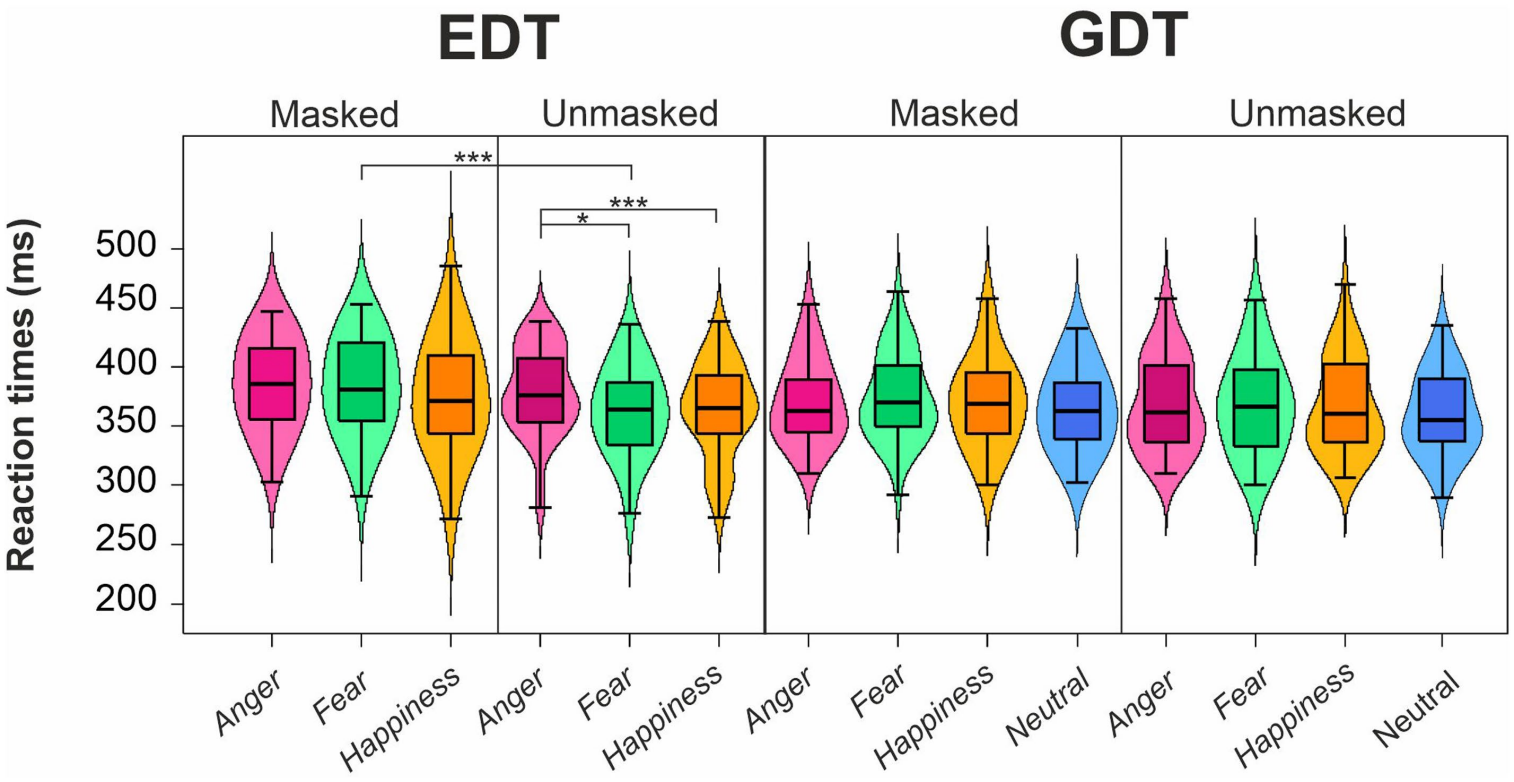
Effects of emotional facial expressions on reaction times in the Emotional (EDT) and Gender Discrimination Task (GDT). Boxplots are reported inside violin plots, which depict kernel probability density. The black line of the boxplot represents the median of the data, and the lower and the upper box’s boundaries indicate the first and third quartiles, respectively. The violin plot width shows the data frequency. Black dots represent outliers. * *p* < 0.05, *** *p* < 0.0001.

The four-way ANOVA on mean RTs of go trials (Figure 3; Table 2) revealed two main effects. First, the factor Emotion showed a main effect as participants’ RTs were longer for angry (376.44 ± 38.25 ms) than fearful (372.57 ± 39.43 ms) and happy faces (369.54 ± 41.80 ms). Second, we found an effect of Mask demonstrating that participants took longer to respond to masked (377.02 ± 40.08 ms) than unmasked facial expressions (368.68 ± 39.30 ms).

**Table 2:** Statistical analysis results of go trials reaction times (RTs).

Four-way parametric ANOVA on RTs Within-Participant Factors: Emotion (3 levels: Anger, Fear, Happiness); Task (2 levels: EDT, GDT); Mask (2 levels: Mask, No Mask) Between-Participant Factor: DA (2 levels: high, low)								
Effect			Value of parameters	*p-*values	*M*_diff_	95% CI	Effect size	BF_10_
*Main*	DA		F(1, 38) = 1.84	0.183	–	–	0.05	0.74
	Task		F(1, 38) = 0.65	0.425	–	–	0.02	1.25
	**Emotion**		**F(1.89, 71.99) = 11.87**	**<0.0001**	–	–	**0.24**	**0.50**
	**Post-hoc comparisons of the main effect of Emotion**							
	**Anger vs. Fear**		**t(38) = 3.08**	**0.011**	**3.87**	**[1.04; 6.70]**	**0.21**	**2.92**
	**Anger vs. Happiness**		**t(38) = 4.79**	**0.0001**	**6.90**	**[3.98; 9.82]**	**0.37**	**>100**
	Fear vs. Happiness		t(38) = 1.96	0.173	–	–	0.16	0.62
	**Mask**		**F(1, 38) = 38.62**	**<0.0001**	**8.34**	**[5.79; 10.89]**	**0.50**	**88.81**
*Two-way interaction*	DA * Task		F(1, 38) = 0.34	0.566	–	–	0.01	0.50
	DA * Emotion		F(1.89, 71.99) = 1.69	0.194	–	–	0.04	0.06
	DA * Mask		F(1, 38) = 1.08	0.306	–	––	0.03	0.16
	Task * Emotion		**F(1.99, 75.49) = 9.81**	**<0.001**	–	–	**0.20**	**0.48**
	Post-hoc comparisons of the interaction effect Task * Emotion							
	EDT:	**Anger vs. Fear**	**t(38) = 4.74**	**<0.001**	**8.86**	**[4.85; 12.88]**	**0.49**	**>100**
		**Anger vs. Happiness**	**t(38) = 6.05**	**<0.0001**	**12.56**	**[8.32; 16.80]**	**0.66**	**>100**
		Fear vs. Happiness	t(38) = 1.66	0.634	–	–	0.19	0.50
	GDT:	Anger vs. Fear	t(38) = −0.61	1.00	–	–	−0.07	0.15
		Anger vs. Happiness	t(38) = 0.65	1.00	–	–	0.07	0.15
		Fear vs. Happiness	t(38) = 1.18	1.00	–	–	0.12	0.22
	Task * Mask		**F(1, 38) = 10.77**	**0.002**	–	–	**0.22**	**1.15**
	Post-hoc comparisons of the interaction effect Task * Mask							
	Mask:	EDT vs. GDT	t(38) = 1.49	0.573	–	–	0.23	1.99
	No Mask:	EDT vs. GDT	t(38) = 0.05	1.00	–	–	0.01	0.10
	EDT:	**Mask vs. No Mask**	**t(38) = 5.58**	**<0.0001**	**13.05**	**[9.17; 16.94]**	**0.61**	**>100**
	GDT:	Mask vs. No Mask	t(38) = 2.41	0.083	–	–	0.21	1.22
	Emotion * Mask		**F(2.00, 75.99) = 5.42**	**0.006**	–	–	**0.12**	**0.18**
	Post-hoc comparisons of the interaction effect Emotion * Mask							
	Mask:	Anger vs. Fear	t(38) = −0.42	1.00	–	–	−0.05	0.14
		Anger vs. Happiness	t(38) = 2.40	0.194	–	–	0.28	2.21
		Fear vs. Happiness	t(38) = 2.65	0.105	–	–	0.30	3.77
	No Mask:	**Anger vs. Fear**	**t(38) = 5.06**	**<0.0001**	**8.65**	**[4.54; 12.75]**	**0.47**	**>100**
		**Anger vs. Happiness**	**t(38) = 4.45**	**<0.001**	**8.58**	**[4.45; 12.71]**	**0.46**	**>100**
		Fear vs. Happiness	t(38) = −0.04	1.00	–	–	−0.004	0.12
	Anger:	Mask vs. No Mask	t(38) = 2.10	0.382	–	–	0.25	1.41
	Fear:	**Mask vs. No Mask**	**t(38) = 6.82**	**<0.0001**	**13.59**	**[9.26; 17.93]**	**0.70**	**>100**
	Happiness:	Mask vs. No Mask	t(38) = 2.92	0.053	–	–	0.32	5.08
*Three-way interaction*	DA * Task * Emotion		F(1.99, 75.59) = 1.82	0.169	–	–	0.05	0.10
	DA * Task * Mask		F(1, 38) = 2.77	0.104	–	–	0.07	0.31
	DA * Emotion * Mask		F(2.00, 75.99) = 0.78	0.463	–	–	0.02	0.06
	Task * Emotion * Mask		F(1.88, 71.49) = 1.86	0.165	–	–	0.05	0.11
*Four-way interaction*	DA * Task * Emotion * Mask		F(1.88, 71.49) = 1.66	0.199	–	–	0.04	0.22

Three-way parametric ANOVA on RTs Within-Participant Factors: Emotion (3 levels: Anger, Fear, Happiness); Mask (2 levels: Mask, No Mask) Between-Participant Factor: DA (2 levels: high, low)								
Effect			Value of parameters	*p-*values	*M*_diff_	95% CI	Effect size	BF_10_
*Main*	DA		F(1,38) = 1.95	0.171	–	–	0.05	0.65
	Emotion		F(2.64, 100.44) = 0.52	0.648	–	–	0.01	0.01
	**Mask**		**F(1,38) = 11.14**	**0.002**	**3.94**	**[1.29; 6.60]**	**0.23**	**6.29**
*Two*–*way interaction*	DA * Emotion		**F(2.64, 100.44) = 3.29**	**0.029**			**0.08**	**1.89**
	Post-hoc comparisons of the interaction effect DA * Emotion							
	Anger	High vs. Low	t(38) = 1.32	1.00	–	–	0.29	1.02
	Fear	High vs. Low	t(38) = 1.14	1.00	–	–	0.23	0.68
	Happiness	High vs. Low	t(38) = 1.98	0.872	–	–	0.41	5.69
	Neutral	High vs. Low	t(38) = 1.04	1.00	–	–	0.21	0.57
	High	Anger vs. Fear	t(38) = −0.06	1.00	–	–	−0.01	0.23
		Anger vs. Happiness	t(38) = −0.95	1.00	–	–	−0.15	0.24
		Anger vs. Neutral	t(38) = 0.54	1.00	–	–	0.09	0.24
		Fear vs. Happiness	t(38) = −0.85	1.00	–	–	−0.12	0.24
		Fear vs. Neutral	t(38) = 0.69	1.00	–	–	0.11	0.24
		Happiness vs. Neutral	t(38) = 1.94	0.954	–	–	0.31	0.25
	Low	Anger vs. Fear	t(38) = −0.80	1.00	–	–	−0.12	0.24
		Anger vs. Happiness	t(38) = 1.87	1.00	–	–	0.33	0.29
		Anger vs. Neutral	t(38) = −0.51	1.00	–	–	−0.10	0.24
		Fear vs. Happiness	t(38) = 2.51	0.261	–	–	0.41	0.35
		Fear vs. Neutral	t(38) = 0.19	1.00	–	–	0.03	0.23
		Happiness vs. Neutral	t(38) = −3.04	0.067	–	–	−0.37	0.32
	DA * Mask		**F(1,38) = 12.73**	**<0.001**			**0.25**	**25.27**
	Post-hoc comparisons of the interaction effect DA * Mask							
	Mask	High vs. Low	t(38) = 1.04	1.00	–	–	0.32	1.15
	No Mask	High vs. Low	t(38) = 1.72	1.00	–	–	0.53	28.28
	High	Mask vs. No Mask	t(38) = −0.16	1.00	–	–	−0.02	0.12
	**Low**	**Mask vs. No Mask**	**t(38) = 4.88**	**<0.0001**	**8.15**	**[4.21; 12.10]**	**0.46**	**>100**
	Emotion * Mask		F(2.74, 104.23) = 0.55	0.631	–	–	0.01	0.07
*Three-way interaction*	DA * Emotion * Mask		F(2.74, 104.23) = 0.92	0.425	–	–	0.02	0.13

EDT, Emotional Discrimination Task; GDT, Gender Discrimination Task; DA, Delta Arousal, which is the index of the arousal difference between emotions (see text for more details); Effect size = partial eta squared (η^2^ₚ) for the ANOVAs and Cohen’s d for the post-hoc tests; BF_10_ = Bayes Factors report the ratio of likelihood of the alternative hypothesis to the likelihood of the null hypothesis; *p*-values were reported in bold when < 0.05; alpha level in post-hoc (i.e., pairwise) comparisons were adjusted according to Bonferroni correction. Differences in the estimated marginal means (*M*_diff_) are reported along with their 95% confidence interval (CI).

Both main effects were qualified by the interaction Emotion*Mask. Post-hoc comparisons indicated that participants displayed significantly longer RTs for angry unmasked expressions (374.42 ± 38.99 ms) than fearful (365.77 ± 38.71 ms) and happy unmasked faces (365.85 ± 40.05 ms). In addition, masked fearful expressions (379.37 ± 39.21 ms) elicited longer RTs than unmasked fearful expressions (365.77 ± 38.71 ms). Crucially, we also found an interaction Emotion*Task. Post-hoc comparisons indicated that in the EDT, RTs to angry faces (382.50 ± 37.99 ms) were longer than those to fearful (373.64 ± 40.33 ms) and happy expressions (369.95 ± 44.58 ms), whereas no such difference was observed in the GDT. Finally, there was also an interaction Task*Mask. Post-hoc comparisons revealed that during EDT, participants had longer RTs for masked (381.89 ± 42.69 ms) than unmasked faces (368.84 ± 38.79 ms).

The control three-way ANOVA on mean RTs of go trials in the GDT showed a main effect of Mask, as participants responded slower to the masked (372.31 ± 37.12 ms) than unmasked expressions (368.37 ± 39.33 ms). We also found two interactions involving the factor Delta Arousal. The first one was the Delta Arousal*Mask. Post-hoc comparisons indicated that participants of the Low Delta Arousal subgroup were slower for masked (366.38 ± 32.03 ms) than unmasked faces (358.22 ± 34.51 ms). The second interaction was Delta Arousal*Emotion. However, no comparison survived to Bonferroni’s correction.

### 3.2 Movement times

**Figure 4:**
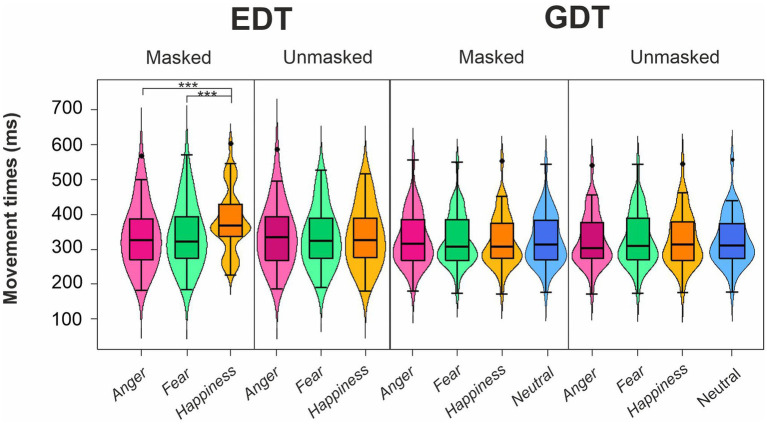
Effects of emotional facial expressions on movement times in the Emotional (EDT) and Gender Discrimination Task (GDT). All conventions are as in Figure 3.

The four-way ANOVA on mean MTs of go trials (Figure 4; Table 3) revealed three main effects. First, the main effect of the Task was that participants had longer MTs in the EDT (344.54 ± 88.77 ms) than in GTD (326.74 ± 75.27 ms). Second, the main effect of Emotion was due to the fact that MTs for happy expressions (342.59 ± 84.67 ms) were longer than those for angry (332.57 ± 82.38 ms) and fearful expressions (331.75 ± 81.07 ms). Third, the main effect of Mask revealed that masked faces (339.48 ± 84.57 ms) had significantly longer MTs than unmasked faces (331.79 ± 80.76 ms).

**Table 3:** Statistical analysis results of go trials movement times (MTs).

Four-way parametric ANOVA on MTs Within-Participant Factors: Emotion (3 levels: Anger, Fear, Happiness); Task (2 levels: EDT, GDT); Mask (2 levels: Mask, No Mask) Between-Participant Factor: DA (2 levels: high, low)								
Effect			Value of parameters	*p-*values	*M*_diff_	95% CI	Effect size	BF_10_
*Main*	DA		F(1,38) = 0.02	0.897	**–**	**–**	< 0.001	0.76
	**Task**		**F(1,38) = 7.22**	**0.011**	**17.80**	**[11.76; 23.85]**	**0.16**	**>100**
	**Emotion**		**F(1.40, 53.31) = 26.08**	**< 0.0001**	**–**	**–**	**0.41**	**50.07**
	**Post-hoc comparisons of the main effect of Emotion**							
	Anger vs. Fear		t(38) = 0.80	1.00	**–**	**–**	0.06	0.12
	**Anger vs. Happiness**		**t(38) = −4.92**	**0.0001**	**−10.02**	**[−14.51; −5.53]**	**−0.35**	**>100**
	**Fear vs. Happiness**		**t(38) = −6.09**	**<0.0001**	**−10.85**	**[−15.11; −6.58]**	**−0.40**	**>100**
	**Mask**		**F(1,38) = 28.92**	**< 0.0001**	**7.69**	**[4.54; 10.83]**	**0.432**	**12.57**
*Two-way interaction*	DA * Task		F(1,38) = 0.00	0.970	**–**	**–**	< 0.001	0.12
	DA * Emotion		F(1.40, 53.31) = 0.41	0.592	**–**	**–**	0.01	0.03
	DA * Mask		F(1,38) = 0.39	0.537	**–**	**–**	0.01	0.12
	Task * Emotion		**F(1.55, 59.07) = 35.38**	**< 0.0001**	**–**	**–**	**0.48**	**> 100**
	Post-hoc comparisons of the interaction effect Task * Emotion							
	EDT:	Anger vs. Fear	t(38) = 1.16	1.00	**–**	**–**	0.13	0.24
		**Anger vs. Happiness**	**t(38) = −5.91**	**< 0.0001**	**−20.51**	**[−28.58; −12.43]**	**−0.56**	**> 100**
		**Fear vs. Happiness**	**t(38) = −7.12**	**< 0.0001**	**−22.50**	**[−29.85; −15.15]**	**−0.68**	**> 100**
	GDT:	Anger vs. Fear	t(38) = −0.29	1.00	**–**	**–**	−0.03	0.13
		Anger vs. Happiness	t(38) = 0.33	1.00	**–**	**–**	0.04	0.13
		Fear vs. Happiness	t(38) = 0.52	1.00	**–**	**–**	0.07	0.15
	Task * Mask		**F(1,38) = 46.58**	**< 0.0001**	**–**	**–**	**0.551**	**82.09**
	Post-hoc comparisons of the interaction effect Task * Mask							
	Mask:	**EDT vs. GDT**	t(38) = 3.85	**0.002**	**26.38**	**[17.20; 35.57]**	**0.52**	**> 100**
	No Mask:	EDT vs. GDT	t(38) = 1.39	0.692	**–**	**–**	0.22	1.50
	EDT:	**Mask vs. No Mask**	t(38) = 6.78	**<0.0001**	**16.26**	**[10.66; 21.87]**	**0.52**	**> 100**
	GDT:	Mask vs. No Mask	t(38) = −0.73	1.00	**–**	**–**	−0.08	0.15
	Emotion * Mask		**F(1.50, 57.18) = 34.56**	**<0.0001**	**–**	**–**	**0.48**	**33.11**
	Post-hoc comparisons of the interaction effect Emotion * Mask							
	Mask:	Anger vs. Fear	t(38) = 0.15	1.00	**–**	**–**	0.02	0.12
		**Anger vs. Happiness**	**t(38) = −6.35**	**<0.0001**	**−19.93**	**[−27.76; −12.10]**	**−0.57**	**> 100**
		**Fear vs. Happiness**	**t(38) = −7.16**	**<0.0001**	**−20.15**	**[−27.76; −12.54]**	**−0.59**	**> 100**
	No Mask:	Anger vs. Fear	t(38) = 1.12	1.00	**–**	**–**	0.11	0.19
		Anger vs. Happiness	t(38) = −0.06	1.00	**–**	**–**	−0.01	0.12
		Fear vs. Happiness	t(38) = −1.02	1.00	**–**	**–**	−0.12	0.22
	Anger:	Mask vs. No Mask	t(38) = 0.41	1.00	**–**	**–**	0.05	0.14
	Fear:	Mask vs. No Mask	t(38) = 1.50	1.00	**–**	**–**	0.15	0.28
	Happiness:	**Mask vs. No Mask**	**t(38) = 6.85**	**<0.0001**	**20.50**	**[12.60; 28.39]**	**0.58**	**> 100**
*Three-way interaction*	DA * Task * Emotion		F(1.55, 59.07) = 0.17	0.788	**–**	**–**	0.00	0.02
	DA * Task * Mask		F(1,38) = 0.51	0.481	**–**	**–**	0.01	0.75
	DA * Emotion * Mask		F(1.50, 57.18) = 0.40	0.611	**–**	**–**	0.01	0.11
	Task * Emotion * Mask		**F(1.56, 59.10) = 39.44**	**< 0.0001**	**–**	**–**	**0.51**	**> 100**
	Post-hoc comparisons of the interaction effect Task * Emotion * Mask							
	EDT Mask:	Anger vs. Fear	t(38) = −0.41	1.00	**–**	**–**	−0.06	0.18
		**Anger vs. Happiness**	**t(38) = −7.32**	**<0.0001**	**−42.62**	**[−54.27; −30.96]**	**−1.17**	**> 100**
		**Fear vs. Happiness**	**t(38) = −7.30**	**<0.0001**	**−41.71**	**[−53.18; −30.25]**	**−1.16**	**> 100**
	EDT No Mask:	Anger vs. Fear	t(38) = 1.93	1.00	**–**	**–**	0.30	0.91
		Anger vs. Happiness	t(38) = 0.55	1.00	**–**	**–**	0.09	0.20
		Fear vs. Happiness	t(38) = −1.59	1.00	**–**	**–**	−0.25	0.55
	GDT Mask:	Anger vs. Fear	t(38) = 0.81	1.00	**–**	**–**	0.13	0.23
		Anger vs. Happiness	t(38) = 1.56	1.00	**–**	**–**	0.25	0.53
		Fear vs. Happiness	t(38) = 0.78	1.00	**–**	**–**	0.12	0.23
	GDT No Mask:	Anger vs. Fear	t(38) = −1.02	1.00	**–**	**–**	−0.23	0.46
		Anger vs. Happiness	t(38) = −1.45	1.00	**–**	**–**	−0.16	0.28
		Fear vs. Happiness	t(38) = 0.10	1.00	**–**	**–**	0.02	0.17
	EDT Anger:	Mask vs. No Mask	t(38) = −0.17	1.00	**–**	**–**	−0.03	0.17
	EDT Fear:	Mask vs. No Mask	t(38) = 2.30	0.65	**–**	**–**	0.37	1.85
	**EDT Happiness:**	**Mask vs. No Mask**	**t(38) = 7.65**	**<0.0001**	**43.81**	**[32.33; 55.30]**	**1.22**	**> 100**
	GDT Anger:	Mask vs. No Mask	t(38) = 1.12	1.00	**–**	**–**	0.18	0.31
	GDT Fear:	Mask vs. No Mask	t(38) = −0.99	1.00	**–**	**–**	−0.16	0.27
	GDT Happiness:	Mask vs. No Mask	t(38) = −1.47	1.00	**–**	**–**	−0.23	0.47
	Anger Mask:	EDT vs. GDT	t(38) = 1.61	1.00	**–**	**–**	0.26	0.57
	Fear Mask:	EDT vs. GDT	t(38) = 1.80	1.00	**–**	**–**	0.29	0.76
	**Happiness Mask:**	**EDT vs. GDT**	**t(38) = 6.46**	**<0.0001**	**55.88**	**[38.61; 73.15]**	**1.03**	**> 100**
	Anger No Mask:	EDT vs. GDT	t(38) = 1.80	1.00	**–**	**–**	0.29	0.76
	Fear No Mask:	EDT vs. GDT	t(38) = 0.89	1.00	**–**	**–**	0.14	0.25
	Happiness No Mask:	EDT vs. GDT	t(38) = 1.31	1.00	**–**	**–**	0.21	0.38
*Four-way interaction*	DA * Task * Emotion * Mask		F(1.56, 59.10) = 0.29	0.696	**–**	**–**	0.01	0.07

Three-way parametric ANOVA on MTs Within-Participant Factors: Emotion (3 levels: Anger, Fear, Happiness); Mask (2 levels: Mask, No Mask) Between-Participant Factor: DA (2 levels: high, low)							
**Effect**		**Value of parameters**	***p-*values**	***M***_**diff**_	**95% CI**	**Effect size**	**BF**_**10**_
*Main*	DA	F(1,38) = 0.01	0.907	–	–	< 0.001	0.73
	Emotion	F(2.74, 104.22) = 0.13	0.927	–	–	0.004	0.02
	Mask	F(1,38) = 1.00	0.324	–	–	0.03	0.24
*Two-way interaction*	DA * Emotion	F(2.74, 104.22) = 0.25	0.846	–	–	0.01	0.07
	DA * Mask	F(1,38) = 0.22	0.638	–	–	0.01	0.18
	Emotion * Mask	F(2.55, 96.94) = 1.44	0.240	–	–	0.04	0.15
*Three-way interaction*	DA * Emotion * Mask	F(2.55, 96.94) = 0.30	0.795	–	–	0.01	0.00

EDT, Emotional Discrimination Task; GDT: Gender Discrimination Task; DA: Delta Arousal, which is the index of the arousal difference between emotions (see text for more details); Effect size = partial eta squared (η^2^ₚ) for the ANOVAs and Cohen’s d for the post-hoc tests; BF_10_ = Bayes Factors report the ratio of likelihood of the alternative hypothesis to the likelihood of the null hypothesis; *p*-values were reported in bold when < 0.05; alpha level in post-hoc (i.e., pairwise) comparisons were adjusted according to Bonferroni correction. Differences in the estimated marginal means (*M*_diff_) are reported along with their 95% confidence interval (CI).

The interactions qualified the main effects. The interaction Task*Emotion revealed that in the EDT, happy faces (358.87 ± 90.22 ms) elicited longer MTs than angry (338.37 ± 88.70 ms) and fearful faces (336.38 ± 86.72 ms). The interaction Task*Mask was due to two effects. First, in the EDT, participants showed longer MTs in response to masked (352.67 ± 91.47 ms) than to unmasked expressions (336.41 ± 85.60 ms). Second, considering only the masked version of the faces, participants had longer MTs in the EDT (352.67 ± 91.47 ms) than the GDT (326.29 ± 75.15 ms). The interaction Emotion*Mask was also due to two effects. First, in the masked condition, participants had longer MTs to happy (352.84 ± 87.17 ms) than to angry (332.91 ± 83.23 ms) and fearful expressions (332.69 ± 82.72 ms). Second, MTs for happy masked faces (352.84 ± 87.17 ms) were longer than happy unmasked faces (332.35 ± 81.34 ms).

All these effects were further qualified by the triple interaction Emotion*Task*Mask. Post-hoc comparisons unveiled that only in the masked conditions of the EDT, happy expressions (380.78 ± 89.70 ms) elicited longer MTs than angry (338.17 ± 89.97 ms) and fearful faces (339.07 ± 90.41 ms). In addition, happy masked expressions had longer MTs in the EDT than in the GDT (324.90 ± 75.81 ms). Finally, in the EDT, happy masked faces (380.78 ± 89.70 ms) had longer MTs than happy unmasked expressions (336.97 ± 86.36 ms).

The control ANOVA on MTs in the GDT did not yield significant effects.

### 3.3 Omission error rates

**Figure 5:**
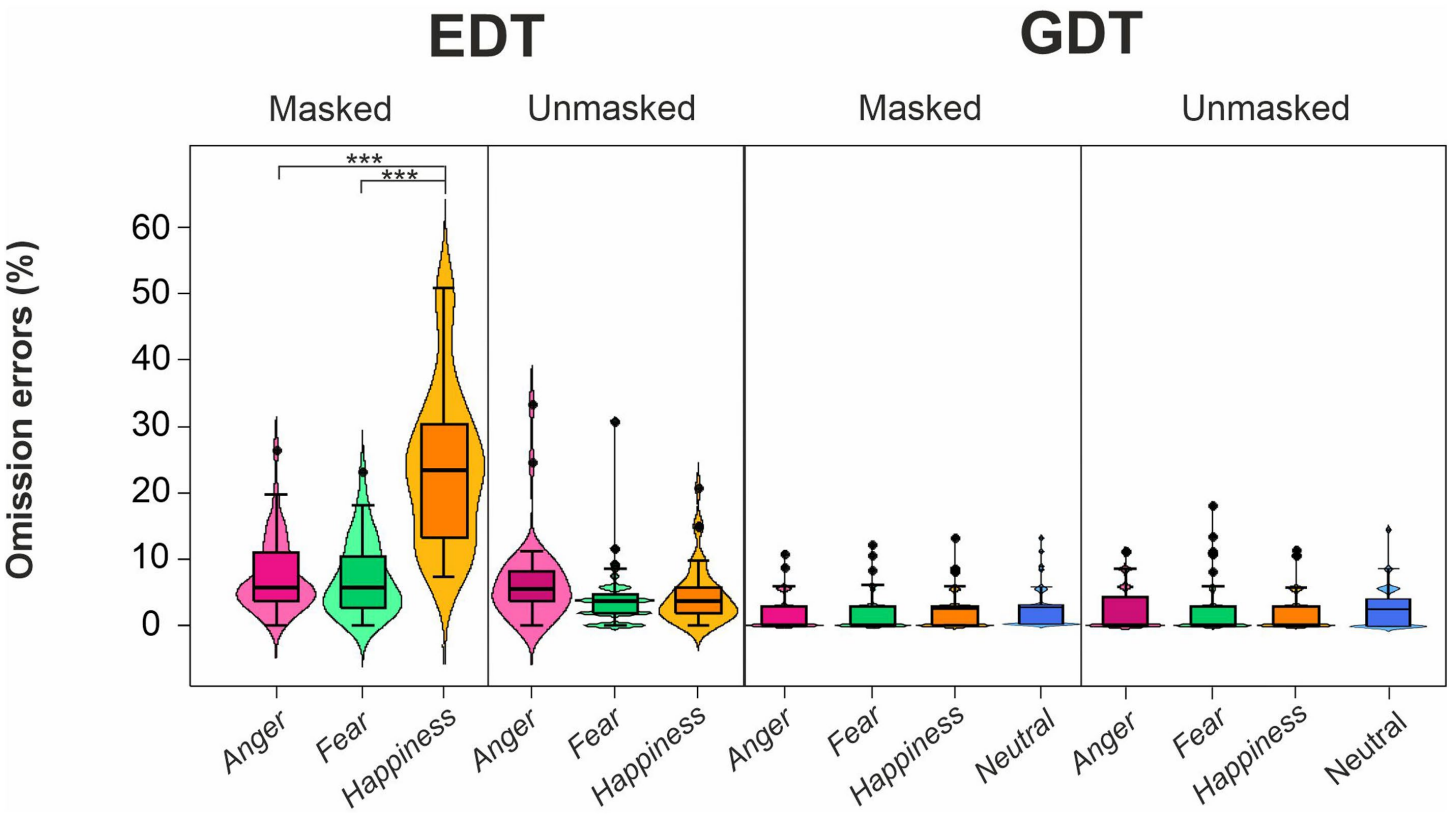
Effects of emotional facial expressions on omission error rates in the Emotional (EDT) and Gender Discrimination Task (GDT). All conventions are as in Figure 3.

The four-way ANOVA on mean OERs (Figure 5; Table 4) unveiled three main effects, i.e., Task, Emotion, and Mask. The Task’s main effect was due to higher OER in the EDT (8.84 ± 9.66) than in the GTD (2.43 ± 3.37). The main effect of Emotion indicated that participants made more OERs for happy (8.29 ± 11.20) than angry (4.58 ± 5.23) and fearful faces (4.03 ± 4.97). Finally, the main effect of the Mask showed that participants had a higher OER in the masked (7.48 ± 9.79) than in the unmasked condition (3.79 ± 4.76).

**Table 4:** Statistical analysis results of omission error rates (OERs).

Four-way parametric ANOVA on OERs Within-Participant Factors: Emotion (3 levels: Anger, Fear, Happiness); Task (2 levels: EDT, GDT); Mask (2 levels: Mask, No Mask) Between-Participant Factor: DA (2 levels: high, low)								
Effect			Value of parameters	*p-*values	*M*_diff_	95% CI	Effect size	BF_10_
*Main*	DA		F(1,38) = 0.33	0.570	**–**	**–**	0.01	0.17
	**Task**		**F(1,38) = 47.32**	**<0.0001**	**6.42**	**[5.08; 7.76]**	**0.55**	**>100**
	**Emotion**		**F(1.49, 56.77) = 45.81**	**<0.0001**	**–**	**–**	**0.55**	**>100**
	**Post-hoc comparisons of the main effect of Emotion**							
	Anger vs. Fear		t(38) = 1.64	0.325	**–**	**–**	0.12	0.28
	**Anger vs. Happiness**		t(38) = −6.28	**<0.0001**	**−3.71**	**[−5.30; −2.13]**	**−0.37**	**>100**
	**Fear vs. Happiness**		t(38) = −8.68	**<0.0001**	**−4.27**	**[−5.71; −2.82]**	**−0.46**	**>100**
	**Mask**		**F(1,38) = 70.62**	**<0.0001**	**3.69**	**[2.52; 4.86]**	**0.650**	**>100**
*Two-way interaction*	DA * Task		F(1,38) = 0.41	0.528	**–**	**–**	0.01	0.49
	DA * Emotion		F(1,49, 56.77) = 0.13	0.818	**–**	**–**	0.00	0.04
	DA * Mask		F(1,38) = 0.31	0.580	**–**	**–**	0.01	0.17
	Task * Emotion		**F(1.63, 62.03) = 45.68**	**<0.0001**	**–**	**–**	**0.55**	**>100**
	Post-hoc comparisons of the interaction effect Task * Emotion							
	EDT:	Anger vs. Fear	t(38) = 2.11	0.251	**–**	**–**	0.25	1.26
		**Anger vs. Happiness**	t(38) = −6.45	**<0.0001**	**−7.30**	**[−10.21; −4.40]**	**−0.56**	**>100**
		**Fear vs. Happiness**	t(38) = −9.94	**<0.0001**	**−8.61**	**[−11.05; −6.16]**	**−0.78**	**>100**
	GDT:	Anger vs. Fear	t(38) = −0.50	1.00	**–**	**–**	−0.05	0.14
		Anger vs. Happiness	t(38) = −0.38	1.00	**–**	**–**	−0.04	0.13
		Fear vs. Happiness	t(38) = −0.20	1.00	**–**	**–**	0.02	0.12
	Task * Mask		**F(1,38) = 104.41**	**<0.0001**	**–**	**–**	**0.73**	**>100**
	Post-hoc comparisons of the interaction effect Task * Mask							
	Mask:	**EDT vs. GDT**	t(38) = 9.16	**<0.0001**	**10.50**	**[8.34; 12.66]**	**0.88**	**>100**
	No Mask:	**EDT vs. GDT**	t(38) = 2.70	**0.042**	**2.33**	**[1.09; 3.56]**	**0.34**	**64.16**
	EDT:	**Mask vs. No Mask**	t(38) = 10.24	**<0.0001**	**7.78**	**[5.79; 9.78]**	**0.70**	**>100**
	GDT:	Mask vs. No Mask	t(38) = −1.10	1.00	−0.39	[−1.07; 0.29]	−0.10	0.19
	Emotion * Mask		**F(1.53, 57.99) = 85.53**	**<0.0001**	**–**	**–**	**0.69**	**>100**
	Post-hoc comparisons of the interaction effect Emotion * Mask							
	Mask:	Anger vs. Fear	t(38) = 0.18	1.00	**–**	**–**	0.02	0.12
		**Anger vs. Happiness**	**t(38) = −8.86**	**<0.0001**	**−8.48**	**[−11.10; −5.87]**	**−0.72**	**>100**
		**Fear vs. Happiness**	**t(38) = −11.45**	**<0.0001**	**−8.57**	**[−10.94; −6.20]**	**−0.80**	**>100**
	No Mask:	Anger vs. Fear	t(38) = 2.46	0.168	**–**	**–**	0.24	1.06
		Anger vs. Happiness	t(38) = 2.03	0.447	**–**	**–**	0.22	0.78
		Fear vs. Happiness	t(38) = 0.08	1.00	**–**	**–**	0.01	0.12
	Anger:	Mask vs. No Mask	t(38) = 0.41	1.00	**–**	**–**	0.04	0.13
	Fear:	Mask vs. No Mask	t(38) = 2.13	0.355	**–**	**–**	0.21	0.69
	Happiness:	**Mask vs. No Mask**	**t(38) = 11.65**	**<0.0001**	**9.74**	**[7.00; 12.48]**	**0.79**	**>100**
*Three-way interaction*	DA * Task * Emotion		F(1.63, 62.03) = 2.59	0.094	**–**	**–**	0.06	0.43
	DA * Task * Mask		F(1,38) = 3.72	0.061	**–**	**–**	0.09	0.67
	DA * Emotion * Mask		F(1.53, 57.99) = 0.26	0.713	**–**	**–**	0.01	0.08
	Task * Emotion * Mask		**F(1.70, 64.72) = 49.00**	**<0.0001**	**–**	**–**	**0.56**	**>100**
	Post-hoc comparisons of the interaction effect Task * Emotion * Mask							
	EDT Mask:	Anger vs. Fear	t(38) = 0.29	1.00	**–**	**–**	0.05	0.18
		**Anger vs. Happiness**	**t(38) = −8.73**	**<0.0001**	**−16.44**	**[−20.22; −12.67]**	**−1.39**	**>100**
		**Fear vs. Happiness**	**t(38) = −11.35**	**<0.0001**	**−16.70**	**[−19.64; −13.76]**	**−1.82**	**>100**
	EDT No Mask:	Anger vs. Fear	t(38) = 3.15	0.077	**–**	**–**	0.49	10.21
		Anger vs. Happiness	t(38) = 2.01	1.00	**–**	**–**	0.31	0.97
		Fear vs. Happiness	t(38) = −0.64	1.00	**–**	**–**	−0.10	0.21
	GDT Mask:	Anger vs. Fear	t(38) = −0.14	1.00	**–**	**–**	−0.02	0.17
		Anger vs. Happiness	t(38) = −1.11	1.00	**–**	**–**	−0.17	0.28
		Fear vs. Happiness	t(38) = −0.88	1.00	**–**	**–**	−0.14	0.24
	GDT No Mask:	Anger vs. Fear	t(38) = −0.60	1.00	**–**	**–**	−0.09	0.20
		Anger vs. Happiness	t(38) = 0.55	1.00	**–**	**–**	0.09	0.20
		Fear vs. Happiness	t(38) = 0.87	1.00	**–**	**–**	0.14	0.24
	EDT Anger:	Mask vs. No Mask	t(38) = 1.31	1.00	**–**	**–**	0.20	0.36
	EDT Fear:	Mask vs. No Mask	t(38) = 3.29	0.053	**–**	**–**	0.52	15.49
	EDT Happiness:	**Mask vs. No**	**t(38) = 11.63**	**<0.0001**	**19.27**	**[15.95; 22.59]**	**1.86**	**>100**
	GDT Anger:	Mask vs. No Mask	t(38) = −0.96	1.00	**–**	**–**	−0.15	0.26
	GDT Fear:	Mask vs. No Mask	t(38) = −1.33	1.00	**–**	**–**	−0.21	0.39
	GDT Happiness:	Mask vs. No Mask	t(38) = 0.38	1.00	**–**	**–**	0.06	0.18
	Anger Mask:	**EDT vs. GDT**	**t(38) = 5.13**	**<0.001**	**5.31**	**[3.22; 7.40]**	**0.81**	**>100**
	Fear Mask:	**EDT vs. GDT**	**t(38) = 4.59**	**0.001**	**4.97**	**[2.80; 7.13]**	**0.73**	**>100**
	Happiness Mask:	**EDT vs. GDT**	**t(38) = 10.22**	**<0.0001**	**21.23**	**[17.06; 25.40]**	**1.63**	**>100**
	Anger No Mask:	EDT vs. GDT	t(38) = 3.21	0.065	**–**	**–**	0.48	8.75
	Fear No Mask:	EDT vs. GDT	t(38) = 0.96	1.00	**–**	**–**	0.15	0.26
	Happiness No Mask:	EDT vs. GDT	t(38) = 2.53	0.377	**–**	**–**	0.40	2.91
*Four-way interaction*	DA * Task * Emotion * Mask		F(1.70, 64.72) = 0.06	0.919	**–**	**–**	0.00	0.07

Three-way parametric ANOVA on OERs Within-Participant Factors: Emotion (3 levels: Anger, Fear, Happiness); Mask (2 levels: Mask, No Mask) Between-Participant Factor: DA (2 levels: high, low)							
Effect		Value of parameters	*p-*values	*M*_diff_	95% CI	Effect size	BF_10_
*Main*	DA	F(1,38) = 1.70	0.200	**–**	**–**	0.04	0.58
	Emotion	*F*(2.83,107.61) = 0.19	0.893	**–**	**–**	0.01	0.01
	Mask	F(1,38) = 0.79	0.380	**–**	**–**	0.02	0.20
*Two-way interaction*	DA * Emotion	F(2.83,107.61) = 1.86	0.143	**–**	**–**	0.05	0.22
	DA * Mask	F(1,38) = 1.28	0.266	**–**	**–**	0.03	0.38
	Emotion * Mask	*F*(2.49, 94.52) = 0.79	0.483	**–**	**–**	0.02	0.08
*Three-way interaction*	DA * Emotion * Mask	F(2.49, 94.52) = 0.71	0.522	**–**	**–**	0.02	0.16

EDT, Emotional Discrimination Task; GDT, Gender Discrimination Task; DA, Delta Arousal, which is the index of the arousal difference between emotions (see text for more details); Effect size = partial eta squared (η2ₚ) for the ANOVAs and Cohen’s d for the post-hoc tests; BF10 = Bayes Factors report the ratio of likelihood of the alternative hypothesis to the likelihood of the null hypothesis; *p*-values were reported in bold when < 0.05; alpha level in post-hoc (i.e., pairwise) comparisons were adjusted according to Bonferroni correction. Differences in the estimated marginal means (*M*_diff_) are reported along with their 95% confidence interval (CI).

These main effects were qualified by two-way interactions, i.e., Task*Emotion, Task*Mask, and Emotion*Mask. Post-hoc comparisons for the Task*Emotion interactions revealed that in the EDT, the OERs were higher for happy (14.15 ± 13.14) than angry (6.84 ± 5.91) and fearful faces (5.54 ± 5.550). The Task*Mask interaction was because, first, in the EDT, the OERs were higher for masked (12.73 ± 11.30) than unmasked expressions (4.95 ± 5.39). Second, participants made more OERs in the EDT than the GDT both in the masked condition (EDT: 12.73 ± 11.30; GDT: 2.23 ± 3.00) and in the unmasked condition (EDT: 4.95 ± 5.39; GDT: 2.62 ± 3.70). The Emotion*Mask interaction was explained by the fact that first, the OERs were higher for happy (13.17 ± 13.71) than angry (4.68 ± 5.15) and fearful faces (4.60 ± 5.11). Second, participants had higher OERs to happy masked than happy unmasked expressions (3.42 ± 4.03).

The triple interaction Emotion*Task*Mask further explained the results. The post-hoc analysis revealed that in the EDT, participants made more OERs in response to masked happy (23.78 ± 11.80) than masked angry (7.34 ± 5.59) and masked fearful faces (7.08 ± 5.60). In addition, in the EDT, the OERs were higher for happy masked faces than for happy unmasked faces (4.51 ± 4.51). Finally, participants had higher OERs in the EDT than GDT for each masked emotion (anger: 2.02 ± 2.82; fear: 2.11 ± 3.00; happiness: 2.55 ± 3.23).

The control ANOVA on the OERs in the GDT did not yield significant effects.

## 4 Discussion

For the first time, we investigated the impact of surgical masks employed during the COVID-19 pandemic on the phenomenon of task-relevance of facial emotional stimuli. In our experiment, we gave the same participants two tasks where the same stimuli consisting of a pseudorandom mix of masked and unmasked facial expressions were shown. In one task, the EDT, participants had to respond according to the stimuli’ valence, whereas in the other, the GDT, they had to respond according to the posers’ gender. We found that responses to emotional stimuli were influenced only in the EDT, i.e., when the emotional content of the stimuli was relevant, but not in the GDT, i.e., when valence was task-irrelevant. Thus, in line with our previous evidence (Mirabella, 2018; Mancini et al., 2020, 2022; Calbi et al., 2022; Mirabella et al., 2023; Montalti and Mirabella, 2023), we showed that only when the emotional content was relevant for providing the correct responses it also affected the behavioral reactions of participants. However, responses to facial emotions in the EDT markedly differ between unmasked and masked conditions. On the one hand, as previously shown (Mancini et al., 2020; Mirabella et al., 2023), participants responded slower to unmasked angry faces than to unmasked fearful and happy faces in the EDT. On the other hand, surgical masks left responses to angry faces unaltered with respect to the unmasked version of the stimuli but impacted fearful and, more prominently, happy expressions. In particular, the RTs to masked fearful faces increased, and those to masked happy faces became more variable, so the difference between these three facial emotions observed in the unmasked condition disappeared. In addition, masked happy faces elicited longer MTs and higher OERs than masked fearful and angry faces.

Relevantly, our results depend not on the stimuli’ arousal but on their valence. Furthermore, significant results have large effect sizes and high values of BF_10_, whereas key non-significant results have values of BF_10_ supporting null hypotheses. Thus, our findings are statistically very solid.

### 4.1 The impact of task-relevance on unmasked faces

As expected, in the unmasked condition, we found that the task-relevance of angry faces matters. In fact, participants showed higher RTs to angry than to happy and fearful unmasked faces in the EDT, whereas no differences in RTs between the three emotional expressions or between emotional and neutral faces appear in the GDT. This evidence aligns with previous results (Mancini et al., 2020; Mirabella et al., 2023), suggesting that angry faces hold participants’ attention more strongly than happy and fearful faces. However, there are also a few important differences with previous research. First, in this experiment, fearful unmasked faces did not elicit longer RTs than happy unmasked faces. Second, the OERs of angry unmasked faces were only nominally but not yet significantly higher than those of happy and fearful unmasked faces (Table 1) as in Mancini et al. (2020). Third, in contrast to the findings of Mancini et al. (2020), our results revealed that angry faces did not significantly increase the length of the MTs than other emotional faces. We suggest that such differences stem from the increased cognitive demands associated with the current task than prior ones. Our rationale is based on the outcomes we previously observed. In Mirabella (2018), we presented fearful and happy faces, along with neutral expressions in the EDT. We found the RTs for the former stimuli increased by approximately 15 ms than the latter. Additionally, we observed significantly higher OERs for fearful faces than for happy faces. In Mancini et al.’s study (2020) study, we increased the task difficulty by including three emotional expressions, i.e., angry, fearful, and happy faces, along with a neutral one. Under these conditions, the difference in RTs in the EDT between fearful and happy faces remained significant but decreased to 9.5 ms, and the OERs were no longer different between the two emotional expressions. In the current study, we dramatically heightened cognitive demands by presenting the masked and unmasked versions of three emotional expressions along with the masked and unmasked versions of the neutral stimuli. In such context, it is highly probable that only the most salient emotional expression, i.e., the angry unmasked faces can hold participants’ attention, prolonging the responses. This aligns well with the notion that the task-relevance phenomenon is contingent on the automatic allocation of attention to threatening stimuli in the EDT driven by the implicit need to assess whether these stimuli may pose a potential threat (Mirabella, 2018; Mancini et al., 2020, 2022; Mirabella et al., 2023; Montalti and Mirabella, 2023). However, in situations where attentional resources are more extensively utilized, a shift in cognitive strategy becomes imperative. This adjustment is necessary as these resources are essential for responding accurately to the various stimuli presented in the task.

### 4.2 The impact of the surgical mask on behavioral reactions to emotional expressions

The surgical mask exhibited several effects in the EDT; yet, it did not impact responses in the GDT, except for one aspect unrelated to valence, as discussed in the Section 4.3. Essentially, masks selectively and profoundly influenced experimental conditions where the stimuli’ valence was essential for giving the correct response as opposed to when participants based their responses on the posers’ gender. When participants responded to the masked stimuli, they had longer RTs, MTs, and higher OERs than for unmasked stimuli. However, these effects widely varied on the emotional expressions. First, reactions to angry expressions remained unaffected, aligning with evidence indicating that surgical masks did not hinder the recognition of angry faces (Grahlow et al., 2022; Proverbio and Cerri, 2022; Rinck et al., 2022; Gil and Le Bigot, 2023; Proverbio et al., 2023). Second, fearful masked faces increased the RTs with respect to unmasked stimuli, while they had no impact on MTs and OERs. This suggests that, in line with Eisenbarth and Alpers (2011), the mouth, not just the eyes, plays a significant role in fear decoding. This finding contrasts with other studies suggesting that the eyes have a prominent role in fear recognition (Whalen et al., 2001; Morris et al., 2002; Adolphs et al., 2005). Third, the presence of masks had a pronounced impact on happy expressions. Surgical masks resulted in highly variable RTs for happy expressions, nullifying the significant differences between the three facial emotions shown in the unmasked condition. Additionally, they substantially increased OERs and MTs compared to fearful and angry faces. We propose that both effects can be attributed to the covering of the mouth, which specifically interferes with processing information related to happiness. The increased difficulty in recognizing masked happy expressions is well known (Marini et al., 2021; Grahlow et al., 2022; Proverbio and Cerri, 2022; Rinck et al., 2022; Ventura et al., 2023). However, here, we demonstrated for the first time that this effect also occurs when visual signals are employed to guide motor actions. Furthermore, we showed that even MTs are affected. This is noteworthy, considering that modulations in MTs are seldom observed, given that participants typically respond using keypresses or saccadic movements. However, unlike saccadic eye movements, which, once initiated, cannot be corrected, reaching arm movements are not ballistic. Hence, MTs can be affected by the experimental context, as a few other studies have previously shown (Coombes et al., 2005; Hälbig et al., 2011; Esteves et al., 2016; Mancini et al., 2020). The lengthening of MTs was likely because participants in the presence of a happy masked face were uncertain about the valence of the expression and thus paid attention to the faces’ valence even during the execution of the reaching arm movement.

### 4.3 The influence of arousal on behavioral reactions

As it is well known that arousal impacts response modulation (Lundqvist et al., 2014), we incorporated the rating of this dimension of emotional stimuli into our analyses. Consistent with our prior investigations (Mirabella, 2018; Mancini et al., 2020, 2022; Calbi et al., 2022; Mirabella et al., 2023; Montalti and Mirabella, 2023), arousal never showed main effects or interaction with stimuli valence. Consequently, we can affirm that all our outcomes are due solely to the valence of facial expressions. In just one instance, we found that behavioral performance was influenced by arousal. The analyses of the RTs in GDT revealed that participants with lower Delta Arousal exhibited slower responses to masked stimuli compared to unmasked stimuli, in contrast to those with higher Delta Arousal. This finding suggests that individuals perceiving masked stimuli as more arousing displayed heightened reactivity to them.

## 5 Conclusion

This study contributes to understanding how surgical masks, commonly used during the COVID-19 pandemic, impact our reactions to facial emotion processing, highlighting the interplay between task demands, stimuli valence, and stimuli arousal. We found that surgical masks selectively impact behavioral performance to emotional stimuli in the EDT when participants were instructed to respond according to the emotional content of the images. Conversely, there was no effect of valence in the GDT. This evidence provides further support to the phenomenon of task-relevance, indicating that responses to emotional stimuli are not automatic as suggested by the motivational model (Bradley et al., 2001), but they are appraised according to the contextual situation and people’s goals in accordance with the appraisal theories of emotions (Moors and Fischer, 2019; Scherer and Moors, 2019). Notably, the effect of valence on participant responses in the EDT was strikingly different when unmasked with respect to masked emotional stimuli were presented. In line with previous findings (Mancini et al., 2020; Mirabella et al., 2023), in the unmasked condition, participants’ attention was held longer by angry than happy or fearful expressions expression, leading to slower responses to such stimuli. However, masks had a pronounced impact on those emotions whose decoding relies to a certain extent on mouth visibility, i.e., fear and happy expressions. The influence of surgical masks was particularly pronounced on happy expressions, introducing variability in RTs and increasing OERs and MTs. This evidence suggests that when facing masked people, observers are less capable of adequately reacting to happiness.

## Data availability statement

The datasets presented in this study can be found in online repositories. The names of the repository/repositories and accession number(s) can be found below. The datasets for this study can be found in the Open Science Framework at the link https://osf.io/pvut6/.

## Ethics statement

The studies involving humans were approved by Local ethical committee “ASST Spedali Civili” di Brescia (protocol number 4452). The studies were conducted in accordance with the local legislation and institutional requirements. The participants provided their written informed consent to participate in this study. Written informed consent was obtained from the individual(s) for the publication of any potentially identifiable images or data included in this article.

## Author contributions

MM: Data curation, Formal analysis, Investigation, Project administration, Writing – original draft. GM: Conceptualization, Funding acquisition, Methodology, Resources, Software, Supervision, Validation, Writing – review & editing.

## Footnotes

^1^https://www.nimh.nih.gov/research/research-conducted-at-nimh/research-areas/clinics-and-labs/ln/shn/software-projects.shtml (Accessed February 21, 2024).

^2^We did not use the valence ratings to make an index, similar to what was done for arousal because valence is binary, encompassing either positive or negative valenced stimuli. Nonetheless, studies have indicated that anger influences action planning in a distinct manner than fear. This does not come as a surprise, as angry faces convey different social signals compared to fearful faces. Unlike fear, anger poses a direct threat to the observer, prompting immediate action. Consequently, combining behavioral indexes for fearful and angry faces may introduce a potential confounding factor. To avoid this, we considered the three emotional expressions as different levels of the factor Emotions.
